# Lessons from a pilot and feasibility randomised trial in depression (Blood pressure Rapid Intensive Lowering And Normal Treatment for Mood and cognition in persistent depression (BRILiANT mood study))

**DOI:** 10.1186/s40814-015-0042-y

**Published:** 2015-12-22

**Authors:** Kirsty Olsen, Denise Howel, Robert Barber, Gary A. Ford, Peter Gallagher, R. Hamish McAllister-Williams, Jonna Nilsson, John O’Brien, Jennie Parker, Alan Thomas

**Affiliations:** 1 Institute of Neuroscience, Campus for Ageing & Vitality, Newcastle University, Newcastle upon Tyne, NE4 5PL UK; 2 Institute of Health & Society, Baddiley-Clark Building, Newcastle University, Newcastle upon Tyne, NE2 4AX UK; 3 Northumberland, Tyne & Wear NHS Foundation Trust, Older Peoples Mental Health Services, Centre for the Health of the Elderly, Campus for Ageing & Vitality, Newcastle upon Tyne, NE4 6BE UK; 4 Medical Sciences Division, University of Oxford, South Parks Road, Oxford, OX1 3PL UK; 5 Institute of Neuroscience & Newcastle Institute for Ageing, Henry Wellcome Building, Framlington Plane, Newcastle University, Newcastle upon Tyne, NE2 4HH UK; 6 Institute of Neuroscience & Northumberland, Tyne & Wear NHS Foundation Trust, Academic Psychiatry, Wolfson Research Centre, Campus for Ageing & Vitality, Newcastle University, Newcastle upon Tyne, NE5 4LP UK; 7 Aging Research Centre, Karolinska Institutet & Stockholm University, Gävlegatan 16, SE-113 30 Stockholm, Sweden; 8 Department of Psychiatry, University of Cambridge, University of Cambridge School of Clinical Medicine, Level E4, Cambridge Biomedical Campus, Box 189, Cambridge, CB2 0SP UK; 9 Newcastle Clinical Trials Unit, Newcastle University, 1-4 Claremont Terrace, Newcastle upon Tyne, NE2 4AE UK; 10 Institute of Neuroscience & Newcastle University Institute for Ageing, Campus for Ageing & Vitality, Newcastle University, Newcastle upon Tyne, NE4 5PL UK

**Keywords:** Depression, Hypertension, Cognitive, Mood, Recruitment, Randomised controlled trials, Pilot

## Abstract

**Background:**

The blood pressure rapid intensive lowering and normal treatment for mood and cognition in persistent depression (BRILiANT mood study) was devised as a pilot study to investigate the feasibility and safety of intensive blood pressure lowering as treatment for persistent mood and cognitive symptoms in older adults with major depressive disorder and to assess the availability of this population for recruitment. In addition, the relationship between reduced blood pressure and the change in cerebral blood flow and mood was to be investigated.

**Methods:**

A single centre pilot randomised controlled trial (RCT), with two parallel groups of intensive or normal treatment for hypertension, recruiting from primary and secondary care and newspaper advert, with an aim of recruiting 66 participants, was observed in this study. At the end of the recruitment period, in order to explore the reasons for failure to recruit to target, surveys were developed and issued to those involved in recruitment.

**Results:**

Recruitment rates were lower than expected which led to the study being expanded to further areas and opened to self-referral via advertisement. However, because of better management of hypertension due to changes in the UK Quality and Outcomes Framework guidelines for blood pressure treatment, few eligible patients were identified and the study closed at the end of the recruitment period, with 13 participants consenting, but 12 failing screening resulting in one recruited participant.

**Conclusions:**

Overall, the BRILiANT mood study was found not to be feasible, and results suggest that the expected patient population no longer exists. To overcome such recruitment difficulties, a prompt commencement of a study after funding so no relevant care changes occur might help prevent similar problems in future studies. In addition, self-referral, in this case via advertisement in papers, may be a useful tool to increase response rate. When recruiting in primary care, direct access to primary care databases, in a secure and anonymised way, may enable more effective screening. Ultimately, the BRILiANT mood study was shown not to be feasible; this was a useful conclusion from this pilot study.

**Trial Registration:**

ISRCTN 64524251; UKCRN Portfolio No: 13284

## Background

Residual mood and cognitive symptoms commonly persist after acute treatment of major depression [1], and such persistence is clinically important, being linked with higher rates of relapse [2, 3], worse psychological functioning [4] and overall poorer longer term outcomes [5–8]. Whilst factors causing symptom persistence in middle-aged and older adults is not fully understood, there is robust evidence that it is strongly related to cerebrovascular disease [9] and prospective studies of vascular diseases have reported that previous major depression or depressive symptoms predicts a two- to threefold increase in coronary heart disease and stroke disease [9]. White matter hyperintensities (WMH), which are regions of high intensity (lesions) seen in the white matter of the brain in magnetic resonance imaging (MRI) scans, are increased in depression [10]. The burden of WMH predicts persistence [11] and worsening of depressive symptoms [12], as well as incident depressive episodes [12, 13]. The neurocognitive deficits in depression have also been associated with WMH [14–19]. In addition, cognitive impairment in general has been related to vascular risk factors [20], and especially to hypertension, WMH are also strongly related to hypertension [21]. MRI studies have reported reduced cerebral blood flow in older adults [22] with evidence that treatment can increase the blood flow suggesting that the adverse effects may be reversible [22]. Although other biological and psychological factors may be important in major depression, cerebrovascular disease evidenced by the presence of WMH appears to be an important and potentially treatable cause of persistent mood and cognitive symptoms. Hypertension is a well-established risk factor for cognitive decline in older adults [23], and deficits in executive function are those most frequently related to this [20]; it occurs in about 50 % of adults over 50 [24] and of these about half are inadequately treated and so have persistent high blood pressure (BP) [24]. Hypertension causes structural vascular adaptation with remodelling and hypertrophy leading to arteriolar luminal narrowing and wall thickening. This leads to compromised cerebral blood flow (CBF). Evidence suggests that BP lowering to the normal range may reverse these changes, with two studies in hypertension reporting improved CBF at 6 months of BP lowering [25,26] and one found improved cognition as well [25].

Evidence suggests that the use of antihypertensive medication can improve mood, cognition and WMH by lowering blood pressure, perhaps via improved cerebral perfusion [25] and this can be measured in the brain using arterial spin labelling (ASL) [27]. No previous studies have examined BP lowering as a treatment strategy in depression.

Therefore, we planned a pilot and feasibility study (the BRILiANT mood study) as a randomised trial investigating intensive BP lowering as an augmentation strategy in older people with hypertension and major depression with persistent symptoms. The objectives were to assess the feasibility and safety of a treatment intervention, intensive blood pressure lowering as a treatment for persistent mood and cognitive symptoms in older people with major depressive disorder (MDD) and assess the availability of the population for recruitment. Secondary objectives were to investigate the relationship between reduction in BP and change in cerebral blood flow and correlations between cerebral blood flow and mood.

Despite running for its full-intended duration, and concerted efforts made by the study team to improve recruitment, the trial failed to recruit adequate participants, with only one participant enrolled and completing the trial. Reported here is an overview of the study design and recruitment strategy, along with the outcome of surveys conducted to investigate the failure to recruit. The ‘lessons learned’ from this will be discussed, in order to inform recruitment protocols and strategies for similar future trials.

## Methods

### Protocol design

The BRILiANT mood study was designed as a single centre pilot randomised controlled trial with parallel groups of two different antihypertensive treatments in older depressed patients with hypertension. A PROBE (prospective randomised open blinded end-point) design was used. Participants for the study were to be recruited from both primary and secondary care. It was approved by the National Research Ethics Service Committee North East—Newcastle & North Tyneside 2 (REC reference 12/NE/0292) and screening begun in May 2013 for a duration of 16 months.

### Objectives

#### Primary objectives

This study was designed as a pilot trial to assess the feasibility and safety of a treatment intervention, intensive blood pressure lowering as a treatment for persistent mood and cognitive symptoms in older people with major depressive disorder (MDD), and assess the availability of the population for recruitment, in accordance with the documented purpose of a pilot study [28]. Feasibility was to be measured by overall recruitment to the trial and recruitment rate; safety was to be measured by recording of all adverse events in participants and carefully determining their relationship to the intervention to determine if they were suspected adverse reactions and by recording dropout rates during the trial.

#### Secondary objectives

Secondary objectives included investigating the relationship between reduction in BP and change in cerebral blood flow and correlations between cerebral blood flow and mood.

#### Treatment interventions

Participants, who were older people (aged 50–80) with major depressive disorder and persistent mood symptoms, continued with their current antidepressant treatment and were randomised into the intensive or standard blood pressure (BP) lowering groups. Randomisation was conducted by the Newcastle Clinical Trials Unit web-based system in a 1:1 ratio stratified by severity (defined by a dichotomous variable indicating whether the HAM-D score at screening was less than or equal to 20). The use of computer-based blocked randomisation generated a unique treatment number for each participant, with four treatment groups for randomisation. They were treated for 12 weeks with study assessments blind to their treatment allocation. Treatment for the standard group followed current National Institute for Health and Care Excellence (NICE) guidelines: for participants aged <55, an angiotension converting enzyme (ACE) inhibitor, e.g. lisinopril or angiotension II receptor blocker (ARB), e.g. losartan, was initiated and for those aged >55, a calcium channel blocker (CCB) was initiated; the intensive group received initial doses of amlodipine (5 mg) and lisinopril (5 mg) daily, with BP monitored on a fortnightly basis and the doses increased if the BP reading was >130/80 mmHg; bendroflumethazide (2.5 mg) was then added daily, with doxazosin/atenolol/spironolactone added to the treatment regime as needed to lower blood pressure to <130/80 mmHg. All study visits (eight for the intensive group and four for the standard group) took place at the Clinical Ageing Research Unit at the Campus for Ageing and Vitality at Newcastle University. The intensive group had extra visits to increase the BP medication more quickly if the BP target had not been achieved. In addition, an MRI scan and a neuropsychological battery comprising 12 scales were undertaken at baseline and 12 weeks to assess the relationship of cerebral blood flow changes to clinical measures. These two visits lasted for 3 h, with the shorter review visits lasting 30 min each.

#### Outcome measures

The primary outcome measure was the score on the Montgomery-Asbery Depression Rating Scale (MDRS) [29] (completed at baseline, 6 and 12 weeks) at 12 weeks, with secondary outcome measures of change in executive and memory function from baseline to 12 weeks, cerebral blood flow, change in mood, change in 24-h ambulatory blood pressure monitoring from baseline to end of study, remission of depressed mood (defined as MADRS <8) and Clinical Global Impression (CGI) [30] severity and improvement. In addition, subjects completed the Beck Depression Inventory (BDI) [31] every 2 weeks.

#### Statistical issues

The planned sample size was 66 participants, with 33 to be allocated to each group; a 10 % attrition rate was factored into this calculation. This number was based on recommendations for minimum numbers needed for pilot clinical trials with continuous outcomes [32].

As this was a pilot study, the statistical analysis was descriptive in nature, providing estimates of key trial parameters to inform power calculations for a future definitive trial [28]. In addition, correlation coefficients were also to be calculated between blood flow measures and measures of depression, executive function and memory, and multivariate statistical techniques were to be used to explore the possibility of producing a smaller number of composite variables describing neurocognitive function.

### Inclusion and exclusion criteria

Individuals aged 50–80 were sought for this study, with a history of MDD and defined as currently depressed. All participants were to be on a stable single antidepressant therapy and have hypertension (BP >140/90 mmHg) with no changes to any medication within the preceding month and a Mini Mental State Exam (MMSE) [33] score of >23. Potential participants identified with another Axis I DSM-IV disorder (other than an anxiety disorder), harmful use of drugs and/or alcohol in the preceding 12 months, on two or more antihypertensive drugs, dementia, stroke, bipolar or psychotic disorder, renal or hepatic impairment, pregnancy, use of other investigational study drugs, MRI contraindications or previous participation in this study were excluded.

### Recruitment and screening

In all, 20 primary care practices (Table 1) carried out screening for this study, with additional screening undertaken in secondary care psychiatry services within NHS trusts and in six secondary care medical clinics in Newcastle and Gateshead. In addition, local newspaper advertisements were also used to identify potential patients. Screening was a three-step process: the initial screening (taking place in primary and secondary care) consisted of a basic screen using the inclusion/exclusion criteria; the second stage was a more detailed telephone screening to check eligibility; and the final, more detailed screening stage took place at the initial study visit.

**Table 1:** Population distribution across total CCG population and participating GP practices

CCG area	Total population in CCGs	Estimated population % of 50–80-year-olds from total CCG population	Total GP practices in CCGs	No. of participating GP practices	Total practice population from participating GP practices	Estimated population % of 50–80-year-olds from participating GP practices
NHS Durham Dales, Easington and Sedgefield CCG	287,365	51726	42	0	0	0
NHS Gateshead CCG	234,131	35120	35	3	20,711	3107
NHS Hartlepool and Stockton-On-Tees CCG	286,993	45919	42	0	0	0
NHS Newcastle North and East CCG	152,211	18265	18	0	0	0
NHS Newcastle West CCG	130,933	18331	18	1	10,239	1433
NHS North Durham CCG	246,262	41865	32	1	1,663	283
NHS North Tyneside CCG	215,349	34456	29	2	21,058	3369
NHS Northumberland CCG	321,824	61147	46	6	30,967	5884
NHS South Tees CCG	288,784	46205	50	0	0	0
NHS South Tyneside CCG	154,881	26330	30	3	20,209	3840
NHS Sunderland CCG	284,682	45549	55	4	26,389	4222
Total population	2,603,415	424,913	397	20	131,236	22,138

#### Primary care

Research active practices were identified through the UK Primary Care Research Network (PCRN) and sent information about the study. Those practices interested carried out a computer screen of patient lists using all key study entry criteria. Antidepressant therapy was used as a proxy for depression coding, as searches using coding proved problematic. Potential participants identified as meeting the criteria and deemed suitable by their primary care team were then sent an invitation letter from their GP inviting them to contact the study team if they wished to participate. A further telephone screening was then conducted on all respondents to check eligibility (confirmation of age, current medication and mood), and if applicable, a participant information sheet sent to the individual and an appointment made for baseline assessment. If eligible to enter the study, informed written consent was obtained by a member of the study team.

It should be noted that during the course of the study, changes to the structure of the UK National Health Service included the abolition of Primary Care Trusts and the introduction of Clinical Commissioning Groups (CCG), who are clinically led statutory groups responsible for the planning and commissioning of healthcare services for their local area. Although the geographical areas remained virtually unchanged, it did mean a reorganisation of the management systems in place. Some services of CCGs were taken over by the North East Commissioning Support Unit (NECS), who provide support to CCG’s in order to improve patient and healthcare outcomes. In addition to this, changes were introduced to the Quality outcomes Framework (QoF) for the treatment of hypertension in primary care.

As all study visits were being carried out at the Campus for Ageing and Vitality in Newcastle upon Tyne, recruitment was confined to Newcastle, Northumberland, Sunderland and northern areas of the Tees, Esk and Wear Valley Trust. We also added further CCGs: North Durham, Durham Dales, Easington and Sedgefield, during the study, in an attempt to widen the net for recruitment (Table 1).

#### Secondary care

Those in secondary care were screened for potential eligibility using all key study entry criteria by the North East of England Mental Health Research Network (MHRN) and Dementias and neurodegenerative Diseases Research Network (DenDRoN) by examining both paper and computerised clinical notes.

An invitation letter was then sent to the individual by their treating consultant if he/she deemed such an approach appropriate. The same procedure as for primary care was then implemented.

#### Enhancing recruitment

During the course of the study, in order to address difficulties experienced in recruiting participants, additional strategies were implemented. In March 2014, recruitment was broadened to three further CCG areas, so that in total, we had approval to recruit from seven CCGs in the North East England area. In order to approach GPs directly about the study, the chief investigator presented at one GP forum, and the trial manager and clinical support officer from DenDRoN gave short presentations to all GPs separately at another GP forum, which were both held locally. Meetings were also held by the CI and study team with consultant psychiatrists, MHRN and DeNDRoN staff and PCRN/NECS staff. In addition to recruitment from primary care and psychiatry services, attempts were made to identify patients with hypertension through acute hospital clinics (e.g. diabetes, cardiovascular) in Newcastle and Gateshead from December 2013. Finally, direct recruitment from the public via advertisements in regional newspapers was also tried.

#### Advert

Advertisements were placed in three local newspapers: the Chronicle, Journal and Metro, during December 2013, January and March 2014, for a total of 4 weeks. The Evening Chronicle is read by more than 170,000 readers daily, the Metro which is a free paper has a daily circulation of nearly 58,000 and 23,000 copies of the Journal are sold every day. We also took advantage of the Journal Live page, which included a landing page directly to the BRILiANT mood study on the Newcastle Biomedicine webpage; a total of 41 ‘hits’ were recorded. We also used a pilot scheme offered to us without charge by NIHR CRN, using a text-back service. Potential participants were asked to text ‘BPMood’ to a dedicated text number, through which the research team then contacted them.

### Surveys

Following the end of the study recruitment period, the Trial Steering Committee (TSC), which had suggested and supported the above changes in recruitment strategy, requested that the research team sought to explore the reasons for the trial’s failure to recruit enough eligible participants. Questions were developed by the study team, based upon areas of concern that had emerged during the recruitment phase of the study (such as search criteria used, changes to QoF BP guidelines, visibility of the study, participant contact method and engagement of GP practices), using successive iteration which was approved and finalised by the TSC. Different surveys were constructed, in order to investigate the experiences of those services involved (and representative services not involved) in recruitment to this trial. These surveys were sent electronically via SurveyMonkey, along with a study information sheet, to GP practices that screened for the study (19), GP practices who did not (40, matched to the same geographic healthcare (CCG) areas), consultants (17) and MHRN staff who screened for the study (11). All surveys were anonymous and open for responses for 3 weeks.

## Results

### Primary objectives

The trial was found not to be feasible, with only one participant recruited. This participant completed all assessments with no adverse events.

### Secondary objectives

All clinical and imaging data were collected but with only one participant; no analysis was attempted.

### Recruitment

The study recruitment ran from May 2013 to August 2014.

#### Recruitment from primary care

In the CCG areas approved for the study, the total number of potentially available GP practices is 397 with a population of approximately 425,000 of 50–80-year-olds. From this, 20 practices participated with a population of 22,000 (Table 1).

**Fig. 1:**
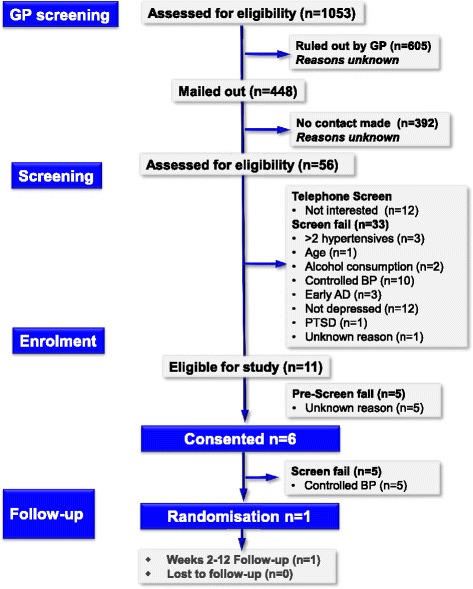
Consort diagram showing screening and recruitment figures from primary care

Screening carried out in primary care (Fig. 1) yielded 1053 potentially eligible participants, of which some were ruled out by the GP: 448 of these were sent an invitation letter from the GP practice. From this, 56 (5 % of those identified) contacted the study team to express interest, and telephone screening was carried out on 51. As a result of the screening, 7 declared no interest in participating and 33 were ineligible. In total, 11 (1 % of those initially identified) were found to fit the eligibility criteria, and from this, 6 were consented into the trial. Following more detailed screening at the initial study visit at the Clinical Ageing Research Unit, further exclusions were made due to either controlled blood pressure, or no current depressed mood; only 1 (about 0.1 % of those identified) was found to be suitable and randomised into the study.

#### Recruitment from secondary care

In areas approved for the study, 26 mental health consultants, from four NHS Trusts, for younger and older adult patients were approached, of whom 19 agreed to participate.

**Fig. 2:**
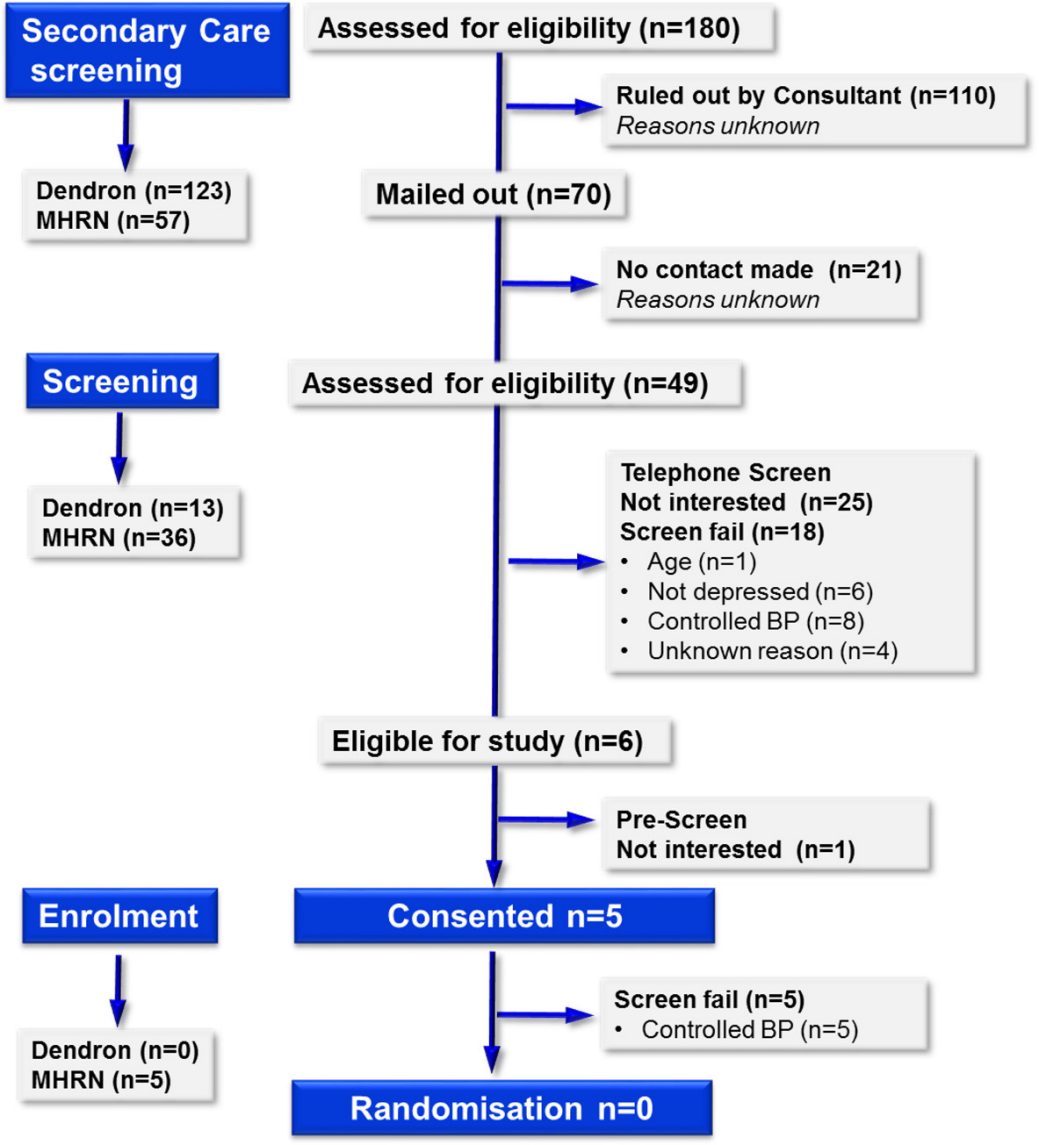
Consort diagram showing screening and recruitment figures from secondary care

In secondary care (Fig. 2), a total of 180 potential participants, of which 123 were identified by DeNDRoN and 57 by the MHRN, were screened, and 70 were contacted. From these, 49 (27 % of those identified) contacted the study team to express interest, with 45 screened via telephone. This resulted in 18 being found to be not eligible and 21 declaring the wish not to proceed further. In total, 6 (3 % of those identified) were found to be eligible, and 5 were consented into the study. As a result of the more detailed screening at study visit 1, none were found to be suitable, and none were randomised into the study.

#### Advert

**Fig. 3:**
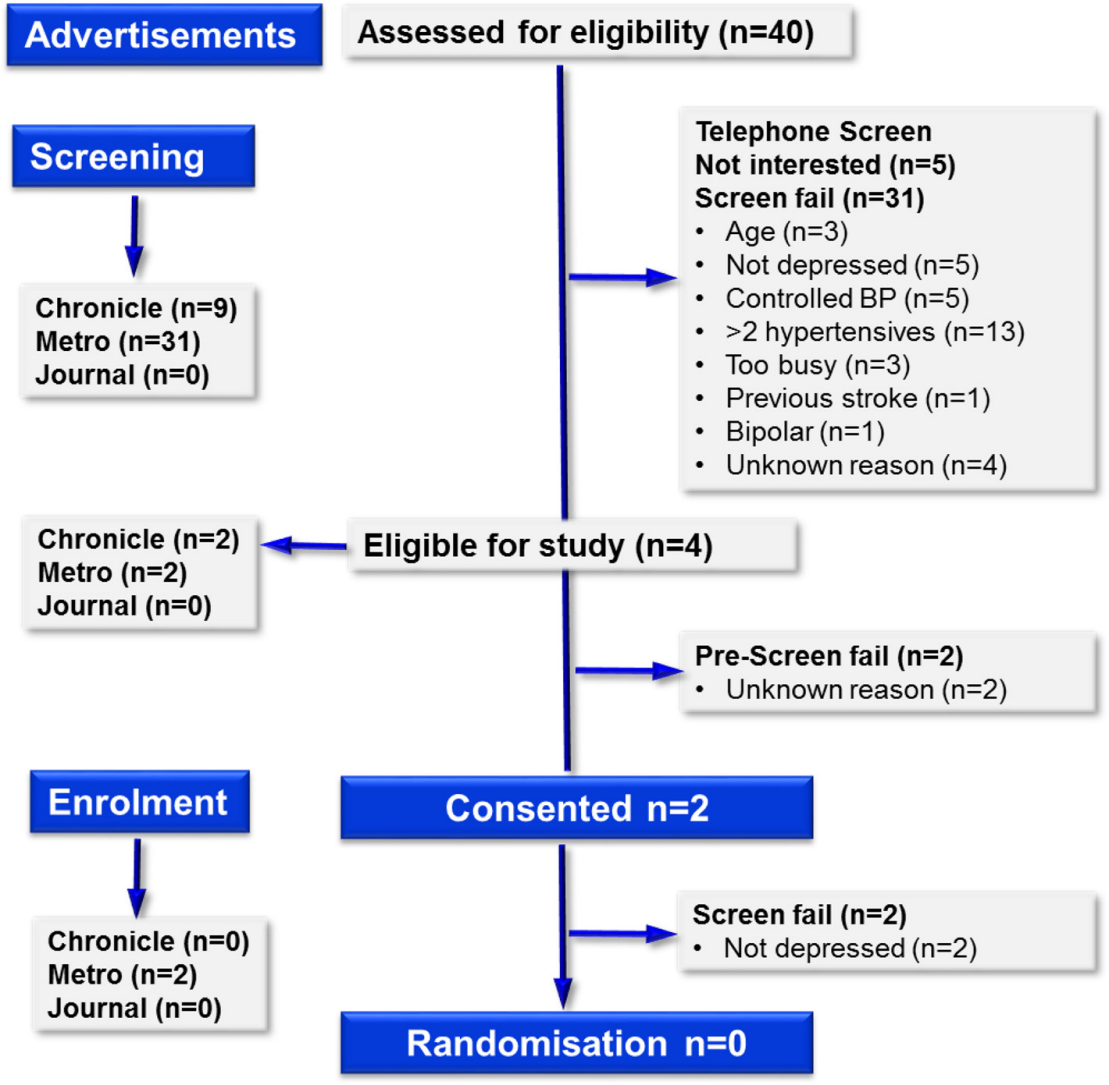
Consort diagram showing screening and recruitment figures from advertisements in local newspapers

As a result of advertising (Fig. 3), 40 people contacted the study team to express interest. From these, 37 were telephone screened, with 31 found not to be eligible and 2 people declaring no further interest. From this recruitment stream, 4 (10 % of those identifying themselves to the team) were found to be eligible, and 2 were consented into the study. As a result of more detailed clinical screening at study visit 1, neither was found to be suitable, and none were randomised into the study.

Overall, from all recruitment streams, 1233 potentially eligible participants were identified from GPs, consultants and self-referrals from the general public, with 151 of these telephone screened. From this, a total of 21 eligible participants were identified; of these, 13 were consented into the study. Ten failed further screening as blood pressure was controlled, two participants failed screening for depression criteria. The study closed at the end of the planned recruitment phase having enrolled one participant (Figs. 1, 2 and 3).

### Surveys to explore reasons for recruitment difficulties

#### GP practices that screened for eligible participants

In all, 10 responses were received from the 19 surgeries contacted (53 % response rate), with one respondent not completing the survey. The responses were received from practice managers and not from GPs themselves. They indicated that the majority (8/9, 89 %) felt the participant search criteria was clear and easy to follow although a small number noted their computer systems caused difficulties, but nothing could have been done differently by the study team to help with identifying patients (100 % of respondents who answered). Most of those who responded stated that they contacted all eligible participants (6/9, 67 %), and the managers felt the changes to the QoF BP guidelines had not made a difference to how patients were treated (7/8, 87.5 %). A wide range of reasons were given for why potentially eligible participants were not contacted, with the patient having another significant illness also present being cited most. The number of visits involved for the participants, and the differing ways a diagnosis of depression is recorded in clinical notes emerged as barriers to screening for this study.

#### GP practices who did not screen for eligible participants

In all, 12 responses were received (30 % response rate). Most of the respondents (67 %) had heard of the study, and of those that had not 3/4 (75 %) indicated that they would have been interested in taking part. Most respondents did not give a reason for why they felt unable to participate, but for those that did, the main reasons cited were there was no time to do research and concern about the burden on the study subjects.

#### MHRN staff who screened for the study

In all, 6 responses were received (55 % response rate). All respondents felt that the search criteria were clear and easy to follow. However, over half (67 %) reported that they had encountered problems when screening, with BP not being recorded (3/5, 60 %) and a small number of people found to be eligible (2/5, 40 %) cited as the main reasons for difficulty identifying study subjects. It was also noted that the number of study visits might have been too onerous.

#### Consultants

In all, 4 responses were received (24 % response rate). All were interested in recommending suitable participants for this study. Two respondents specified why they felt most patients would be excluded from this study, with one citing normal BP and one the restrictive criteria for all intervention studies.

## Discussion

The BRILiANT mood study was developed as a pilot and feasibility randomised controlled trial (RCT) to investigate using rapid blood pressure lowering to alleviate persistent mood and cognitive symptoms in major depressive disorder in older adults. Despite the expectations of an available population (Table 1) based on the then current NICE Clinical Guideline [24], difficulties were experienced in the process of recruitment at several stages. The study team and TSC identified recruitment difficulties and throughout the recruitment period implemented several new strategies to try and improve recruitment. Attempts by the study team to improve recruitment had mixed results. Expanding recruitment areas geographically had little impact on the numbers of eligible participants identified, but the use of direct recruitment via the advert yielded a good response rate. The responses from the surveys suggested that most clinicians were satisfied with the study design, though some felt any clinical trial is too much of a burden e.g. ‘patients do not like to travel and would be put off by the number of visits’. Positively then, the study design itself did not cause significant problems with recruitment; the one eligible subject completed all study assessments without any problems or adverse events, and the search criteria were appropriate. Nevertheless, the fact that several participants identified did not meet the screening criteria, e.g. wrong age and presence of dementia, suggests that the criteria were not applied consistently. In spite of these efforts, the study closed, having recruited only one participant in the planned time period.

Twenty GP practices (about 5 % of available GP practices) agreed to participate in the study (NECS informed the study team that all research active practices were given information about the study; however, they do not maintain records of who declined, or reasons why). This limited support was felt to be a limiting factor in this study. Using ONS and CCG population data, Table 1 shows the population distribution across the CCG areas approved for this study. Based on the office for National Statistics data [34], the total population for the 20 GP practices is approximately 1.5 million, of whom approximately 15 % are aged between 50 and 80 years old (based on the data obtained from Public Health England [35], with the CCG data provided by NECS [36]). This provided us with a potential target population of about 22,000 who were potentially screened by primary care, and of these, 1053 (about 5 %) were identified as potentially eligible by screening (see Fig. 1). These data suggest that at the recruitment rate we achieved (one randomised subject from 5 % of practices), we would only have 20 randomised subjects if we had engaged all GP practices in the North East of England. This suggests that we would have needed to screen from over three times the actual number of patients existing in our target age group in the North East to obtain our 66 subjects, leading to the conclusion that currently such a study is not feasible.

A key question then is whether the target population (50–80-year-olds with inadequately treated hypertension and major depression with residual depressive symptoms) exists and was not identified (a problem with the recruitment process) or does not actually exist. When the study team set out to investigate reasons behind the poor recruitment, it was discovered that there had been important recent changes in the management of blood pressure in primary care at the instigation of the UK Department of Health, which had changed the Quality and Outcomes Framework (QoF) for treatment of hypertension. The QoF is the annual reward and incentive programme in the UK in primary care, linking measures of performance to funding. QoF awards surgeries achievement points for various health outcomes including

- Managing some of the most common chronic diseases, e.g. asthma, diabetes
- Implementing preventative measures, e.g. regular blood pressure checks

As part of the 2013/14 contract changes, the Department of Health (DH) implemented changes to the QOF effective from 1 April 2013 [37]; one of which was the BP threshold was lowered from 150/90 to 140/90 mmHg for patients aged 79 or under, directly affecting the study target population. This was implemented just as the study was about to start. Effectively, primary care was incentivised to identify and treat people with hypertension in the age group the study was trying to recruit from. Although practice managers responding to the survey did not think this as a factor, the fact that five out of six subjects consented from primary care and five out of five from secondary care failed eligibility because of normal blood pressure suggests that better BP management was a significant factor. Although numbers are small, it indicates that most people recorded as hypertensive in NHS records may now have normal blood pressure. The QOF change is likely to have been an important factor in causing recruitment difficulty and suggests that currently in the UK, the target population for this study may not exist in sufficient numbers to make such a study feasible.

A further problem that emerged from the survey responses was the variation in the diagnosis recording. In particular, it was difficult to identify patients with major depressive disorder due to the differing ways it is recorded in clinical notes. In theory searching, using coding as a search parameter could alleviate this; however, with over 400 different relevant codes in use, this is not an easy option to apply. Furthermore, GPs may be reluctant to use coding systems to classify psychological diagnoses [38]. In this study, medication (use of antidepressants) was used as a proxy to try to overcome this barrier and allowed identification of people who were potentially being treated for depression, although many were not depressed when screened, suggesting that as with hypertension, many who were recorded as depressed did not currently have low mood and so searching on the basis of antidepressant medication may not have been an effective enough method to detect those who were symptomatic.

However, there were also process problems in trying to identify potential participants. Recruitment into clinical trials can be problematic [39], particularly for participants with severe mental illness [40], with many factors influencing the decision to participate [41]. A study in the same area at the same time as this one found that it was much more difficult to recruit people from primary care with depression [42] than with a diagnosis of hypertension (although as seen, most would now be treated and not hypertensive). Given the nature of depression, with symptoms such as amotivation and anergia, such problems are not surprising, and we found that less than 10 % of those whom consultants thought were eligible were actually willing to participate. In addition to this, further problems can arise when recruiting participants from primary care, with difficulties with the quality of databases and computer searches and researchers often overestimating the population of patients available for a particular study [43], and GPs excluding potentially eligible participants from trials [44]. In our study, although the study managers stated they thought the search criteria were clear and easy to follow, over 10 % of identified subjects failed telephone screening for reasons which should have been identified by the computerised screening, e.g. wrong age or on two or more antihypertensives (see Fig. 1). This indicates that the criteria were not accurately applied in practice and raises the possibility that potential subjects were also missed. Finally, recruitment using advertising did not yield a significant number of suitable participants [45].

The final notable barrier, which was felt to have contributed to recruitment problems, is that of the time delay experienced between the writing of the grant proposal and the start of the study. This study experienced a delay of 2 years which it took to finalise contracts and obtain relevant statutory approvals, which allowed changes to take place (notably in the QoF guidelines) which affected recruitment. Having identified an expected available population, it seems to have disappeared by the time the study started. When identified potentially eligible participants contacted the study team, then the symptoms were no longer present, resulting in many participants being screened out at various stages (e.g. telephone screening, baseline screening) who had been identified as eligible. Despite concerns that the number of study visits may have been too onerous, this did not deter participants, as most were willing to attend, but failed at screening, with the one recruited participant completing the study suggesting that this might not be the case.

## Conclusions

It appears the patient population required for this study may no longer exist in sufficient numbers. The length of time taken to start this study following funding allowed the QoF changes to take place was likely a contributing factor. In addition, the process of patient identification is cumbersome and inaccurate. Future studies recruiting in primary care would benefit from direct access to primary care databases, in a secure and anonymised way, and could allow for a pre-screening of records to determine the number of potentially eligible participants prior to the start of the study. Direct recruitment via advertisement may be a useful tool to increase response rate; although in this study, large numbers were identified but no one was finally eligible. Overall, although ultimately the BRILiANT mood study was shown not to be feasible, this was a useful conclusion from this pilot study [28].
